# Efficacy and Safety of a DOAC Compared to Unfractionated Heparin and A Low Molecular Weight Heparin in the Prevention of Thromboembolism in Hematopoietic Stem Cell Transplant Recipients

**DOI:** 10.5812/ijpr-143213

**Published:** 2024-02-21

**Authors:** Saeed Azimi, Abbas Hajifathali, Sayeh Parkhideh, Jamshid Salamzadeh, Bardia Rahmati-Kamel, Farzaneh Dastan, Mahshid Mehdizadeh, Mahdiye Abiyarghamsari, Maria Tavakoli-Ardakani

**Affiliations:** 1 Department of Clinical Pharmacy, School of Pharmacy, Shahid Beheshti University of Medical Sciences, Tehran, Iran; 2 Hematopoietic Stem Cell Research Center, Shahid Beheshti University of Medical Sciences, Tehran, Iran; 3 School of Medicine, Tehran University of Medical Sciences, Tehran, Iran; 4 Chronic Respiratory Disease Research Center, National Research Institute of Tuberculosis and Lung Disease, Shahid Beheshti University of Medical Science, Tehran, Iran; 5 Department of Internal Medicines, Loghman Hakim Hospital, Shahid Beheshti University of Medical Sciences, Tehran, Iran; 6 Pharmaceutical Sciences Research Center, Shahid Beheshti University of Medical Sciences, Tehran, Iran

**Keywords:** HSCT, Thrombosis, Apixaban, Dalteparin, Heparin, Prophylaxis

## Abstract

**Background:**

Hematopoietic stem cell transplantation (HSCT) patients are at risk of thromboembolic events, making thromboprophylaxis crucial.

**Objectives:**

This study aimed to compare apixaban, a direct factor Xa inhibitor (DOAC), with dalteparin and unfractionated heparin for thromboprophylaxis in HSCT recipients. The safety outcome included the assessment of hemorrhagic events.

**Methods:**

In this open-label randomized clinical trial, 182 HSCT recipients were divided into three groups: Apixaban (n = 61, 2.5 mg two times a day), dalteparin (n = 59, 5000 IU daily), and unfractionated heparin (n = 62, 5000 IU twice daily). These anticoagulant regimens were administered after central vein catheterization and during hospitalization. The primary clinical outcome was the risk of thrombosis, and the secondary outcome was the rate of bleeding. Relevant laboratory results were analyzed using appropriate statistical tests.

**Results:**

Among the 61 patients in the apixaban group, six experienced thrombosis (9.83%), with four (6.65%) of them on anticoagulants. In the dalteparin group, three patients (5%) developed thrombosis, two of whom (3.38%) were on anticoagulants. In the heparin group, all four thrombosis cases (6.4%) occurred in patients on anticoagulants (P = 0.543 overall and P = 0.776 in anticoagulant users). Only two cases of bleeding were reported (1.09% overall), one in the dalteparin group (1.69%) and the other in the apixaban group (1.63%).

**Conclusions:**

Apixaban, dalteparin, and heparin demonstrated similar effectiveness in preventing thromboembolism in HSCT recipients. Furthermore, the comparison of bleeding rates across the study groups did not reveal significant differences. Larger studies with higher event rates may yield more precise conclusions.

## 1. Background

Hematopoietic stem cell transplantation (HSCT) is a medical procedure used to treat blood malignancies. While HSCT offers benefits such as increasing the life expectancy of patients, it can also lead to complications that worsen the patient's condition during treatment (1, 2). One of these complications is thromboembolic events, which can have various underlying causes (3). Additionally, patients undergoing HSCT may experience hemorrhagic complications, such as diffuse alveolar hemorrhage and hemorrhagic cystitis (4, 5).

Thrombotic events in HSCT recipients can be classified into several categories, including venous thromboembolic event (VTE), catheter-related thrombosis (CRT), sinusoidal obstruction syndrome (SOS) or veno-occlusive disease (VOD), and transplant-associated thrombotic microangiopathy (TA-TMA) (6). Among these, pulmonary thromboembolism (PTE) and deep vein thrombosis (DVT) are the two primary forms of VTE observed in patients with cancer and blood malignancies (7). Venous thromboembolic event is commonly seen in HSCT patients due to various factors, including malignancy, infections, intense conditioning treatments, immunomodulatory regimens, total body radiation, extended periods of immobilization or hospitalization, and the use of central vein catheters (7, 8). The occurrence rate of VTE after HSCT ranges from up to 7.5%, while clinically symptomatic bleeding occurs in a range of 15.2% to 27.1% (8-10).

Catheter-related thrombosis is a complex condition influenced by both patient-specific factors and catheter-related factors. Several factors can contribute to the development of CRT, including malignancy, a history of catheterization, recent surgery, prolonged catheterization duration, anticoagulant therapy, age, diabetes, obesity, chemotherapy, and thrombophilia (11, 12).

The veno-occlusive disease is a severe complication that can occur following a myeloablative conditioning regimen. Patients with this condition typically experience painful enlargement of the liver, fluid retention leading to weight gain, and elevated bilirubin levels (13, 14).

Transplant-associated thrombotic microangiopathy is another life-threatening disorder that can develop within 100 days after transplantation due to endothelial damage related to the treatment type and underlying diseases. Clinical manifestations of this condition include thrombocytopenia, hemolysis, acute renal failure, mental status changes, and organ dysfunction (15, 16).

## 2. Objectives

Guidelines for preventing thrombosis in cancer patients recommend the use of various types of heparins and direct oral anticoagulants (DOACs) (17). Both categories of medications have been investigated in numerous studies to assess their advantages and limitations (18). Low molecular weight heparins offer the benefits of easier dose adjustment and do not require continuous monitoring. However, dose adjustment is necessary in patients with renal failure. Additionally, due to the injectable route of administration, they may be less preferable for patients compared to DOACs (19, 20). On the other hand, DOACs are administered orally, demonstrating a lower risk of recurrent venous thromboembolism (VTE) compared to low molecular weight heparins. However, patients using DOACs tend to experience higher rates of severe bleeding and clinically relevant non-major bleeding (CRNMB) in comparison to those prescribed low molecular weight heparins (21). Despite a review of existing studies in this area, there is a lack of well-documented research comparing the effectiveness of different anticoagulants specifically in hematopoietic stem cell transplant (HSCT) recipients (22). HSCT recipients are particularly susceptible to complications from drug therapy compared to other cancer patients. Therefore, selecting an appropriate anticoagulation regimen can help prevent numerous life-threatening conditions and enhance the quality of life for HSCT recipients. This study aimed to compare the efficacy (thrombosis prevention) and safety (bleeding rate) of apixaban, dalteparin, and unfractionated heparin in HSCT recipients.

## 3. Methods

This study was conducted as an open-label randomized clinical trial at the bone marrow transplant ward of Taleghani Hospital, affiliated with Shahid Beheshti University of Medical Sciences, Tehran, Iran. The study was registered with the codes IR.SBMU.SME.REC.1401.089 and IRCT20100127003210N25 in the Ethics Committee of Shahid Beheshti School of Pharmacy and the Iranian Registry of Clinical Trials (IRCT), respectively.

### 3.1. Patients

Patients admitted to the Bone Marrow Transplant Ward were considered eligible for participation in this study if they met the following inclusion criteria: Aged at least 18 years, weight ≥ 40 kg, Caprini VTE score ≥ 5, a life expectancy of more than 60 days, functional status score of 2 or less according to ECOG criteria, creatinine clearance ≥ 30 mL/min, platelet count ≥ 50,000/µL, liver enzyme levels (alanine transaminase (ALT) and aspartate transaminase (AST)) less than three times the upper normal limit, international normalized ratio (INR) ≤ 1.6, and not pregnant. Patients with any of the following conditions were excluded from the study: Bacterial endocarditis, severe liver disease (Childs Pugh Class B or C cirrhosis), active bleeding, severe hypertension (systolic blood pressure > 170 mmHg and diastolic blood pressure > 110 mmHg), drug interactions, especially with apixaban (concurrent use of carbamazepine, phenytoin, phenobarbital, rifampin, rifapentine, ritonavir, antifungal azoles like itraconazole and voriconazole), and known anticoagulation disorder or thrombocytopenia due to previous heparin use. Additionally, patients with a history of active bleeding within the past two weeks were not included in the study.

### 3.2. Randomization and Patient Allocation

Based on hospital data, the estimated risk of thrombosis was approximately 40% with heparin-based anticoagulant regimens. We anticipated that the studied regimens would reduce the risk of thrombosis to 10%. Assuming α = 0.01, β = 0.1, a power of 90%, and accounting for a 20% dropout rate, the sample size was calculated to be 65 patients for each arm of the study. Patients were randomly assigned to receive either apixaban, dalteparin, or heparin (as the standard group). Block randomization was employed to ensure an equal distribution of major confounding factors across the study arms. Two primary factors used for stratified randomization of eligible patients were the risk of thrombosis based on the Caprini VTE score and the type of bone marrow transplant (allogeneic or autologous).

### 3.3. Study Protocol

As baseline data, the sociodemographic information of the patients, their medical and medication history, and baseline laboratory results, including electrolyte levels, complete blood cell counts, and liver and kidney function tests, were recorded. The risk of thrombosis was assessed for each patient using the Caprini VTE score. According to this criterion, patients with a score ≥ 5 required thromboprophylaxis. After the initial evaluations, prophylactic antithrombotic treatment was initiated immediately after jugular vein catheterization. Catheterization was performed in the operating room by the surgical team. After local anesthesia with 1% lidocaine and disinfection of the skin insertion site with povidone-iodine, a polyurethane double-lumen catheter was inserted into the internal jugular vein using the Seldinger technique (23). The catheter placement was confirmed using X-ray imaging and then secured with sutures and a sterile dressing. The insertion sites were monitored daily for thrombosis, infection, signs of swelling, and pain. Dalteparin (5000 units subcutaneously daily, 7500 units if BMI ≥ 40 kg/m^2^), heparin (5000 units subcutaneously twice daily, 7500 units if BMI ≥ 40 kg/m^2^), or apixaban (2.5 mg orally twice daily) were used for each of the three study groups. According to the protocol designed for the bone marrow transplant ward, the anticoagulant regimen was paused for 24 hours before stem cell collection and then resumed immediately afterward. The length of hospitalization in the ward was considered the follow-up period for the patients. During this time, their conditions and laboratory factors were evaluated daily.

The primary outcome was the incidence of thrombosis (including any thromboembolic events, such as DVT, PTE, or CRT), while the incidence of bleeding (considered as the safety variable) was regarded as the secondary outcome. Prophylactic treatment would be discontinued under certain conditions: If the platelet count dropped to less than 50,000/µL at any point during hospitalization, anticoagulant administration was halted. In the event of bleeding associated with any anticoagulants, patients would receive the necessary procedures to stop the bleeding. If severe bleeding occurred (characterized by a drop in hemoglobin levels of at least 2 g/dL, the need for at least 2 units of packed red blood cell transfusion, or hemorrhage in critical areas, such as the skull, spinal cord, eye, intra-abdomen, heart, joints, and muscles, along with the development of compartment syndrome or life-threatening bleeding), the anticoagulant was discontinued. Thrombosis was assessed based on Doppler ultrasound findings. Data analysis was performed using version 26.0 of the SPSS software.

## 4. Results

**Figure 1.**
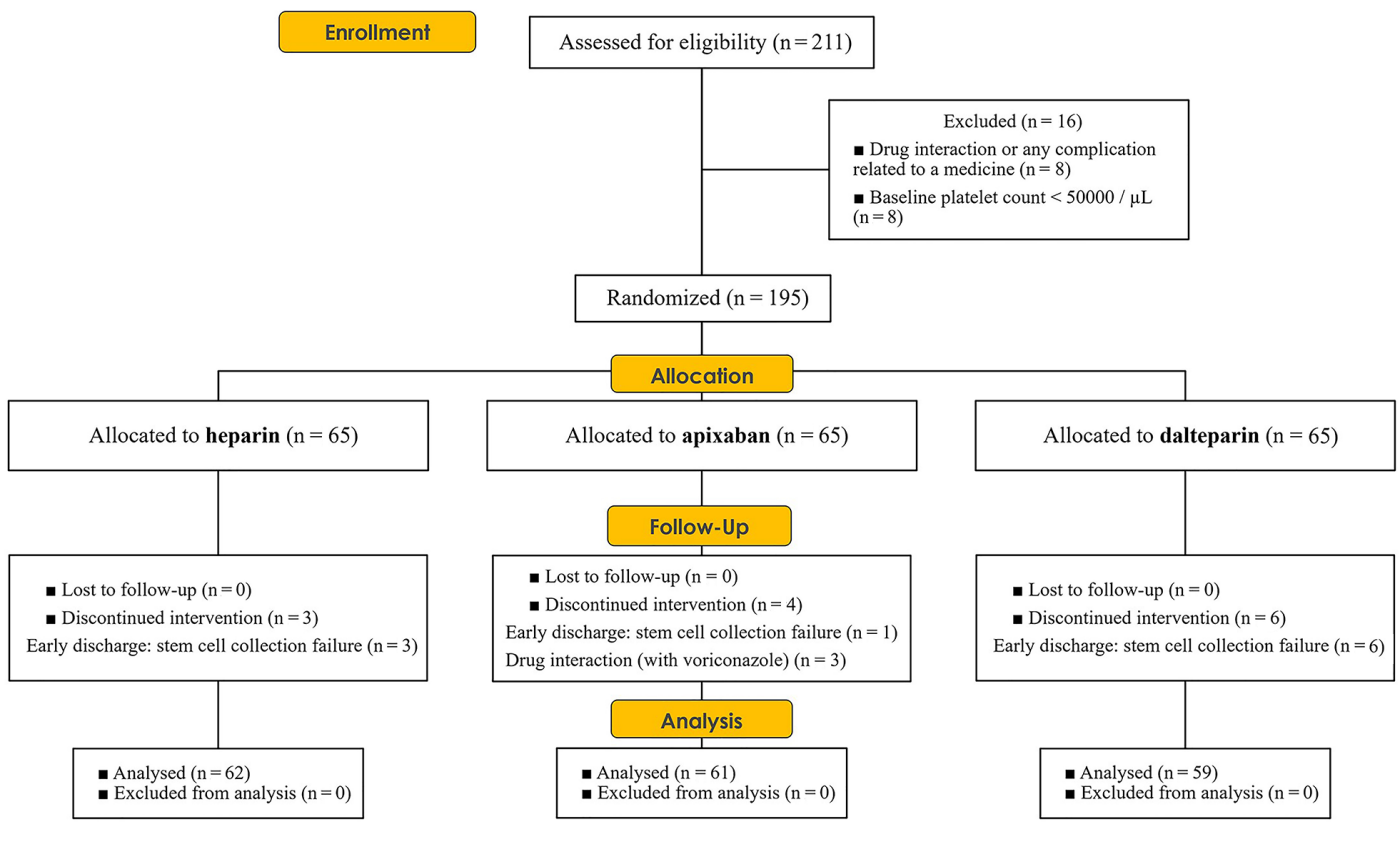
CONSORT diagram: Enrollment, randomization, and allocation of patients participating in the study

The present study was conducted from February 2022 to January 2023. Out of the initial 211 patients, 195 underwent randomization. Sixteen patients were excluded from the study due to baseline platelet counts < 50,000/µL and drug interactions or drug-related complications. Specifically, four patients from the apixaban group, six from the dalteparin group, and three from the heparin group were excluded. The final statistical analysis was conducted on a total of 182 patients (Figure 1). 

Table 1 displays the demographic features and baseline laboratory results of the patients, revealing no significant differences between the groups.

**Table 1.** Demographics and Baseline Laboratory Data of Patients ^a^

Variables	Apixaban (n = 61)	Dalteparin (n = 59)	Heparin (n = 62)	P-Value
**Age (y)**	50.9 ± 10.6	47.8 ± 12.9	47.9 ± 12.6	0.286
**BMI, kg/m** ^**2**^	26.6 ± 4.5	27.3 ± 3.6	26. ± 4.2	0.605
**Gender**				0.547
Male	35	36	32	
Female	26	23	30	
**Hematologic malignancy**				0.869
Multiple myeloma	40	38	35	
Lymphoma	15	14	15	
AML	2	3	6	
ALL	3	2	3	
Other	1	2	3	
**Type of graft**				0.805
Autologous	52	51	51	
Allogenous	9	8	11	
**Comorbidities**				0.287
With comorbidity	25	15	25	
Without comorbidity	36	44	37	
Cardiovascular diseases (hypertension, coronary artery disease, etc.)	9	7	10	
Psychiatric disorders	5	0	3	
Diabetes	2	2	2	
Kidney Diseases	0	1	0	
Liver diseases (hepatitis, cirrhosis, fatty liver, etc.)	0	2	0	
Pulmonary diseases	2	1	2	
Other diseases	7	2	8	
**Caprini VTE score**				0.872
5 - 6	43	41	39	
7 - 8	13	13	18	
> 8	5	5	5	
**Albumin, g/dL**	4.1 ± 0.5	4.1 ± 0.5	4.0 ± 0.6	0.533
**Alp, U/L**	285.2 ± 166.8	279.4 ± 148.8	278.6 ± 158.6	0.969
**ALT, iU/L**	25.0 ± 15.1	28.7 ± 19	31.9 ± 30.3	0.244
**AST, iU/L**	27.1 ± 18	25 ± 12.2	28.4 ± 15.4	0.481
**Direct bilirubin, mg/dL**	0.27 ± 0.12	0.28 ± 0.10	0.27 ± 0.10	0.971
**Total bilirubin, mg/dL**	0.8 ± 0.4	0.8 ± 0.3	0.9 ± 1.6	0.673
**LDH, iU/L**	453.7 ± 352.6	454.95 ± 175.6	493.1 ± 209.4	0.631
**BUN, mg/dL**	14.7 ± 5.5	15.3 ± 5.1	15.1 ± 5.1	0.831
**Cr, mg/dL**	1.08 ± 0.2	1.12 ± 0.2	1.03 ± 0.2	0.132
**Na, meq/L**	140.6 ± 2.8	140.9 ± 3.1	140.8 ± 2.7	0.911
**K, meq/L**	3.9 ± 0.4	4.0 ± 0.5	3.9 ± 0.5	0.412
**RBC, 10** ^**6**^ **/uL**	4.2 ± 0.5	4.1 ± 0.7	4.0 ± 0.5	0.335
**WBC, 10** ^**3**^ **/uL**	12.7 ± 10.2	12.9 ± 8.8	12.8 ± 10.4	0.852
**Hgb, g/dL**	12.3 ± 1.7	12.1 ± 2.0	11.9 ± 1.8	0.496
**HCT, %**	36.5 ± 4.8	36.1 ± 5.3	36.5 ± 4.6	0.865
**Plt, 10** ^**3**^ **/uL**	187.0 ± 58.1	177.2 ± 66.9	177.3 ± 72.8	0.573
**PT, second**	12.2 ± 0.8	13.8 ± 0.3	12.1 ± 0.8	0.512
**PTT, second**	28.2 ± 3.4	29.2 ± 4.6	26.9 ± 8.6	0.505
**INR**	1.01 ± 0.08	1.03 ± 0.07	1.02 ± 0.07	0.656

^a^ Values are expressed as mean ± SD or number.

Table 2 presents the incidence of thrombosis and bleeding in the study groups. In total, 13 cases of thrombosis occurred, with ten patients experiencing thrombosis while on anticoagulant medication. Three patients developed thrombosis when anticoagulant treatment was temporarily halted (due to a platelet count < 50,000/µL). Among the 61 patients in the apixaban group, six had thrombosis (9.83%), with four of them (6.65%) being on anticoagulants. In the dalteparin group, three patients (5%) had thrombosis, and two of them (3.38%) were on anticoagulants. Within the heparin group, all four patients with thrombosis (6.4%) were on anticoagulants. In terms of the incidence of thrombosis, there were no significant differences between the participating groups (P = 0.594 overall and P = 0.776 in cases of thrombosis while on anticoagulants). Only two cases of bleeding were reported during the study: One in the dalteparin group (classified as minor bleeding) and the other in the apixaban group (categorized as CRNMB) (P = 1.00).

**Table 2.** Clinical Outcomes During the Treatment Period ^a^

	Apixaban (n = 61)	Dalteparin (n = 59)	Heparin (n = 62)	Total (n = 182)	P-Value
**Primary outcome**					
Total venous thromboembolism	6 (9.83)	3 (5)	4 (6.4)	13 (7.1)	0.594
Deep vein thrombosis	0 (0.0)	0 (0.0)	0 (0.0)	0 (0.0)	
Pulmonary embolism	0 (0.0)	0 (0.0)	0 (0.0)	0 (0.0)	
Catheter-related thrombosis	6 (9.83)	3 (5)	4 (6.4)	13 (7.1)	
Veno-occlusive disease	0 (0.0)	0 (0.0)	0 (0.0)	0 (0.0)	
Venous thromboembolism on anticoagulant	4 (6.65)	2 (3.38)	4 (6.4)	10 (5.5)	0.776
**Safety outcome**					
Bleeding	1 (1.63)	1 (1.69)	0 (0.0)	2 (1.09)	1.00
Major bleeding ^b^	0 (0.0)	0 (0.0)	0 (0.0)	0 (0.0)	
Clinically relevant non-major bleeding ^c^	1 (1.63)	0 (0.0)	0 (0.0)	1 (0.54)	
Minor bleeding ^d^	0 (0.0)	1 (1.69)	0 (0.0)	1 (0.54)	

^a^ Values are expressed as No. (%).

^b^ Transfusion of red blood cells of two or more units, decrease in hemoglobin level of 2 g/dL or more, involvement of a vital anatomic site, such as retroperitoneal, intracranial, spinal cord, eye, pericardial, articular, or intramuscular compartment syndrome.

^c^ Any sign or symptom of hemorrhage that doesn't fit the ISTH major bleeding criteria but has least one of these features: (1) Needing medical intervention by a healthcare professional; (2) needing hospitalization or more care; (3) leading to a face-to-face visit.

^d^ All non-major bleeding will be considered minor bleeds.

## 5. Discussion

Given the specific conditions of HSCT patients, who may be at risk of thromboembolic events due to their disease and treatment, the use of an anticoagulant regimen can improve their condition if there is an indication. The primary objective of this study was to address this goal. Another aim was to reduce the relatively high rate of thrombosis observed in the bone marrow transplant ward where this study was conducted.

After the initial evaluations, eligible patients received prophylactic anticoagulant regimens, including apixaban, dalteparin, and heparin. The study results indicated that none of these regimens exhibited significant superiority. Additionally, there were no significant differences in terms of bleeding risk associated with these medications.

Due to the limited information available regarding the optimal prevention of thrombosis in HSCT patients, selecting the most appropriate treatment options remains challenging. Most studies in this field focus on treating or preventing thrombosis in cancer patients, particularly those with solid tumors.

The CLOT trial, which employed dalteparin and warfarin, reported thromboembolic events in 8% of patients in the dalteparin group and 15% of patients experienced thromboembolic the dalteparin (n = 336) and warfarin groups (n = 336), respectively (95% confidence interval [CI], 0.30, 0.77, P = 0.002) with the bleeding rate of 6% and 4% for the dalteparin and warfarin groups, respectively (P = 0.090) (24).

In the Hokusai VTE cancer trial, the edoxaban group had a recurrence rate of VTE at 7.9%, compared to 11.3% in the dalteparin group. The bleeding rate was 6.9% in the edoxaban group and 4% in the dalteparin group (95% CI, 0.70, 1.36, P = 0.006 for noninferiority; P = 0.870 for superiority) (25).

The SELECT-D trial found that rivaroxaban had a 4% VTE recurrence rate (95% CI, 2% to 9%), compared to 11% for dalteparin (95% CI, 7% to 16%) at 6, with bleeding rates of 4% and 6% for dalteparin and rivaroxaban, respectively (7).

Similar results were observed in the ADAM VTE trial, where patients on dalteparin had a higher rate of recurrent VTE (6.3%) compared to those on apixaban (0.7%) (P = 0.028), along with a higher incidence of bleeding (1.4% for dalteparin and 0% for apixaban) (P = 0.138) (26).

In the Caravaggio trial, which compared dalteparin and apixaban in treating thromboembolic events in cancer patients, the VTE recurrence rates were 7.9% for dalteparin and 5.6% for apixaban (P < 0.001 for noninferiority). Major bleeding rates were 4% for dalteparin and 3.8% for apixaban, with no significant difference (P = 0.60) (27). Another important factor to consider is the duration and the study population. Most of the patients included in these trials had solid tumor cancers and experienced active thromboembolic events during their treatment. Only seventy patients in the CLOT trial (24), 28 in the SELECT-D trial (7), and 28 patients in the ADAM VTE (26) trial had hematological malignancies. Specific analyses for these subpopulations were not reported. Additionally, some studies did not provide information on central venous catheters (86 patients), except for the CLOT trial. The study duration was six months in all trials, except for the Hokusai VTE cancer trial, which extended to 12 months. Furthermore, the number of patients varied across the studies, ranging from 287 (ADAM VTE) to 1168 (Caravaggio trial) (7, 24-27).

The present study differs in terms of the study population and duration. All participants in the study had hematological malignancies requiring HSCT and received thromboprophylaxis during their hospitalization, with follow-up evaluations conducted during this period. While each patient's hospitalization duration varied, the average durations were reported as 24.6 ± 6.9 days for the apixaban group, 24.8 ± 3.4 days for the dalteparin group, and 25.3 ± 6.1 days for the heparin group.

The primary cause of thrombosis in this study was CRT, with an overall incidence rate of 7.1%, of which 5.5% occurred while patients were on anticoagulant therapy. Some previous studies on CRT in HSCT patients have reported incidence rates ranging from 7.5% to 9%. However, it's worth noting that these studies utilized different methodologies and types of catheters, such as peripherally inserted central catheters (PICCs) (28, 29).

In the AVERT study, which aimed to prevent thromboembolism in cancer patients with central venous catheters, the risk of VTE was lower when apixaban was used (4.8%) compared to a placebo group (18.7%) (P < 0.0001) (30). The lower thrombosis rate in the AVERT study compared to the present study may be attributed to various factors, including differences in the patients' medical conditions and treatment methods.

The medications used in the conditioning regimens for the patients in this study included lomustine, etoposide, cytarabine, melphalan, fludarabine, and busulfan. Additionally, cyclosporin, methotrexate, leucovorin, and anti-thymocyte globulin (ATG) were administered for graft-versus-host disease (GVHD) prophylaxis. It is worth noting that thromboembolic events are known side effects of certain medications, such as cyclosporin, which were part of the treatment regimens.

Furthermore, a significant number of patients with thrombosis had multiple myeloma, and the treatment regimens for these patients often include immunomodulatory agents like thalidomide and lenalidomide, as well as proteasome inhibitors like bortezomib. These agents are known to be associated with an increased risk of thrombosis and microangiopathy (31). Studies have reported that the risk of thrombosis in multiple myeloma patients treated with lenalidomide or thalidomide ranges from 23% to 75%. Thromboprophylaxis has been explored in these patients to reduce thromboembolic events, and among various agents studied (aspirin, warfarin, and low molecular weight heparins), the use of low molecular weight heparins is recommended for high-risk patients (32, 33).

One of the limitations of this study was its small sample size, which was unavoidable due to the specific characteristics of these patients and the limited bed capacity of the hospital ward for hospitalization. Additionally, some patients had to be excluded from the study due to stem cell collection failure.

### 5.1. Conclusions

HSCT patients face potential risks of thromboembolic and bleeding events due to complications and specific treatment conditions. The implementation of treatment and preventive protocols tailored to the patient's clinical situation can help mitigate these treatment-related complications. This study aimed to compare the effectiveness of apixaban, dalteparin, and heparin in thromboprophylaxis for hematopoietic stem cell transplant recipients. The results revealed no significant difference among the three medications, suggesting that the choice of medication may depend on the patient's condition and the physician's judgment. Future studies involving larger populations are expected to explore these considerations further.

## Data Availability

The dataset presented in the study is available on request from the corresponding author during submission or after publication.

## Clinical Trial Registration Code:

IRCT20100127003210N25.
